# Decentralization and health system performance – a focused review of dimensions, difficulties, and derivatives in India

**DOI:** 10.1186/s12913-016-1784-9

**Published:** 2016-10-31

**Authors:** Bhuputra Panda, Harshad P. Thakur

**Affiliations:** 1 Public Health Foundation of India, IIPH-Bhubaneswar, Bhubaneswar, India; 2 School of Health Systems Studies, TISS, Mumbai, India

**Keywords:** Health system performance, Focused review, Decentralization, Dimensions, Difficulties, Derivatives, Efficiency, India, Odisha

## Abstract

**Introduction:**

One of the principal goals of any health care system is to improve health through the provision of clinical and public health services. Decentralization as a reform measure aims to improve inputs, management processes and health outcomes, and has political, administrative and financial connotations. It is argued that the robustness of a health system in achieving desirable outcomes is contingent upon the width and depth of ‘decision space’ at the local level. Studies have used different approaches to examine one or more facets of decentralization and its effect on health system functioning; however, lack of consensus on an acceptable framework is a critical gap in determining its quantum and quality. Theorists have resorted to concepts of ‘trust’, ‘convenience’ and ‘mutual benefits’ to explain, define and measure components of governance in health. In the emerging ‘continuum of health services’ model, the challenge lies in identifying variables of performance (fiscal allocation, autonomy at local level, perception of key stakeholders, service delivery outputs, etc.) through the prism of decentralization in the first place, and in establishing directed relationships among them.

**Methods:**

This focused review paper conducted extensive web-based literature search, using PubMed and Google Scholar search engines. After screening of key words and study objectives, we retrieved 180 articles for next round of screening. One hundred and four full articles (three working papers and 101 published papers) were reviewed in totality. We attempted to summarize existing literature on decentralization and health systems performance, explain key concepts and essential variables, and develop a framework for further scientific scrutiny. Themes are presented in three separate segments of dimensions, difficulties and derivatives.

**Results:**

Evaluation of local decision making and its effect on health system performance has been studied in a compartmentalized manner. There is sparse evidence about innovations attributable to decentralization. We observed that in India, there is very scant evaluative study on the subject. We didn’t come across a single study examining the perception and experiences of local decision makers about the opportunities and challenges they faced. The existing body of evidences may be inadequate to feed into sound policy making. The principles of management hinge on measurement of inputs, processes and outputs. In the conceptual framework we propose three levels of functions (health systems functions, management functions and measurement functions) being intricately related to inputs, processes and outputs. Each level of function encompasses essential elements derived from the synthesis of information gathered through literature review and non-participant observation. We observed that it is difficult to quantify characteristics of governance at institutional, system and individual levels except through proxy means.

**Conclusion:**

There is an urgent need to sensitize governments and academia about how best more objective evaluation of ‘shared governance’ can be undertaken to benefit policy making. The future direction of enquiry should focus on context-specific evidence of its effect on the entire spectrum of health system, with special emphasis on efficiency, community participation, human resource management and quality of services.

## Background

A health care system is a set of activities and actors whose principal goal is to improve health through the provision of public and private medical services [1]. Since the WHO 2000 report, systems’ thinking has re-emerged as the cornerstone for improved health outcomes, and the consequent paradigm shift in policy making from disease-specific initiatives to strengthening of health system. One of the key factors behind this shift was the realization among policy makers that a chronically ill health system would threaten the achievement of millennium development goals (MDG) [2]. Over the years, major global public health institutions have also echoed the views of world health organization (WHO) and started investing in health systems [3–5].

The WHO (2007) explicitly recognized governance as a key pillar of health system building blocks framework. The significance of decentralized governance of health systems as to improve decision making at local levels in different tiers of health service delivery is constantly growing. In India, this has special importance for governments, policy makers and administrators of health services in view of geographic vastness and socio-economic diversities on one hand, and ever-growing health needs and expectation of the population on the other. Further, there is scant empirical evidence examining the effects of decentralization on health system performance, particularly on efficiency and quality of health care services. Therefore, the debate on whether or not decentralization improves equity, efficiency, accountability and quality of services continues to generate curiosity among scholars and policy makers. Assessment of health system performance through macro-level indicators at the national level has only limited value; much more useful information for policy makers could come from sub-regional level (district/institution) assessment of performance, particularly in a country as diverse as India [6–8].

The rationale for undertaking such a focused review is informed by several considerations. First, various development theories, and policies emanating therein, advanced health as a development goal and also created an environment for propagation of alternative policies. Secondly, after the 1978 ‘Alma-Ata’ declaration, governments vowed to ensure community participation towards attainment of health for all. In India, subsequent to the launching of national rural health mission (NRHM), much attention was paid to ‘communization’ through local decision making; but the what’s and how’s of this process required much better understanding [9]. Third, state governments are grappling with issues of poor retention of human resources, adverse fiscal discipline, over-centralized procurement and contracting procedures, erratic supply of drugs and logistics, and low compliance of health service delivery points with pre-defined national standards. Last, many local governments are increasingly facing pressure to introduce reforms, mostly around governance processes.

In this paper, applying a input-process-output framework, we considered service delivery as the ‘existential’ function of the health system, and inputs, processes and outputs as ‘operating domains’ that in our argument dynamically interact with one another and determine the nature and landscape of individual and population health. We attempted i) to examine the dimensions (definitions, functions and instruments; efficiency; quality; health outcomes; conceptual approaches; measuring performance; and tools for measurement) and determinants (health facilities; agents of local decision making; and end-users) of health system performance; ii) discuss the methodological challenges in dealing with performance measurement; and iii) propose derivatives in the form a conceptual framework that is holistic in approach and specific to Indian context.

## Methods

### Search strategy

**Fig. 1:**
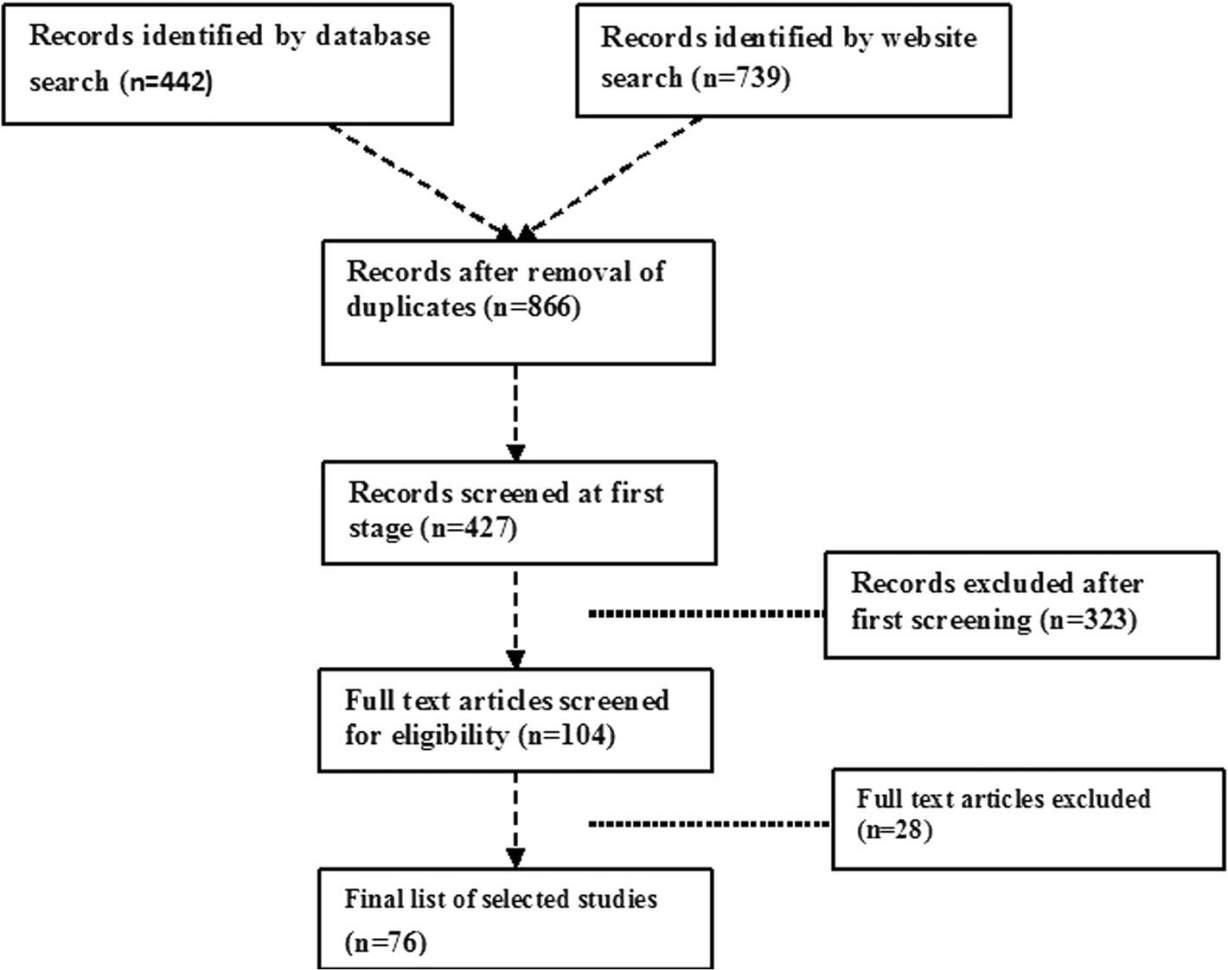
Schematic presentation of search and finalization

This focused, narrative review is based on application of the WHO’s health systems building blocks framework (2000 and 2007) to the principles of management [3, 10]. We used key word search strategy (Fig. 1) for searching literature. A comprehensive computerized search was conducted during April-July 2015 to search for published papers on decentralization and health system performance in general, and in the next level, pertaining to India. The focus was to identify scholarly studies (both qualitative and quantitative) on dimensions of decentralization, difficulties in measuring effects of local self-governance, determinants of health system performance, and to propose derivatives of health system performance in the context of decentralized planning and implementation of health programs. We chose PubMed and Google Scholar database for following reasons: PubMed and Google Scholar are freely accessible. The keyword search with PubMed offers optimal update frequency and includes ‘online first’ articles. PubMed is also an optimal tool in biomedical electronic research. Google Scholar covers a wider journal range, though key word searches were found to be non-specific to commands. PubMed was particularly considered of help both in keyword searching and citation analysis. We also specifically searched the website of World Bank and World Health Organization. Subsequently, the search was narrowed down with use of specific key words. The study was approved by an independent ethics committee of IIPH-Bhubaneswar.

### Key words used during web searching

Health system performance; Health system performance measurement; Decentralization in health; Evaluation of decentralization in health; Evaluation of decentralization; Derivatives of decentralization in health; Dimensions of decentralization in health; Determinants of decentralization in health; Decentralization of health care and its impact on health outcomes.

### Inclusion and exclusion criteria

With use of generic words, such as, ‘decentralization’ and ‘health system performance’, huge volumes of literature were reflected in both the databases. All full texts of articles that focused on ‘dimensions of decentralization in health’, ‘difficulties in measuring/assessing effects of decentralization’, and ‘measurement of health system performance’ were retrieved from the original sources wherever available. Three working papers and 101 published articles were finally selected for full review (*n* = 104). On further scrutiny, 28 articles were excluded at a later stage due to non-conformity with the focus of our study. Publications in English language and covering all geographic areas were included. With respect to time frame, we included studies published from January 1990 till July, 2015. The main reason for taking 1990 as the base year being most of the health sector and economic reforms around decentralization took place in India during early 1990s.

### Data analysis

After conducting full review of the finally selected articles (*n* = 76), we undertook content analysis through systematic text condensation (STC). Emerging themes were explained in four sections: ‘dimensions’, ‘determinants’ and ‘difficulties’ which were summarily derived from the reviewed papers, while the last section on ‘derivatives’ is based on inductive synthesis of literature review along with our observation of rogi kalyan samiti (RKS) governing body meetings at five different health units in Odisha. We audio-recorded the governing body meeting of one district hospital, transcribed and translated it into English; for four other meetings, the researcher retrieved the observations from the field diary. Findings from analysis of RKS meeting observations was used implicitly. Content analysis of these non-participant observations helped in validation of our proposed conceptual framework.

### Definition of ‘key words’ used in searching

#### Dimensions

The dimensions of healthcare performance are those definable, preferably measurable and actionable attributes of the system that are related to its functioning to maintain, restore or improve health [11]. Studies show that appropriate organizational structure promotes productivity, performance, and innovation [12–14]. An appropriate structure should be specified for reforms. It is the organizational structure that determines, organizes, and coordinates all organizational activities [15]. Daft divided organizational structure into two dimensions: structural and contextual [16]. Structural dimensions, which represent internal characteristics, include formalization, centralization, specialization, hierarchy of authority, professionalism, etc. Contextual dimensions are composed of goals, strategies, environment, culture, etc. We assumed that decentralization as a policy reform required structural reforms at organizational level for optimal performance [17].

#### Determinants

Social determinants, such as poverty, migration, gender, and ethnicity have been explored as social determinants of health since they affect the response of the health system and the impact on individual health conditions [18]. We tried to review literature examining influence of decentralization on social determinants of health.

#### Derivatives

Something produced by modification of something preexisting [19].

## Results

### Dimensions

#### Meaning and scope

The key objectives of a health system are to (i) address the health needs of local population and increase access to medicines and treatments for all [20]; and (ii) to expand the reach of health services beyond large cities to the diverse rural areas [21]. It is argued that decentralization can have huge impact on health service functioning, and therefore, has been widely recommended as a reform measure for increasing efficiency in the financing and quality of service delivery which are essentially dimensions of health care system. In other words, The flexibility of decentralized health services is perceived as superior to the rigidities of centralized planning [22].

‘Governance’ focuses on setting the organization’s overall goals, policies and directions, whereas ‘Management’ deals with allocation of resources and overseeing the day-to-day operations of the organization. Governance is visioning while management is realization of those through planned activities. The term ‘decentralization’ is used to denote a variety of power transfer arrangements and accountability systems. Ranging from simple transfer of limited powers to lower management to extensive sectoral reforms, including re-shaping of the provision of even the basic services, all fall within its gambit. The two key elements of decentralization are related to i) the nature and amount of choice that is transferred from central institutions to the peripheral decision making health units (DMHU); and ii) what effect these choices have on the health system performance. In other words, it refers to how the state structure allows sharing of power between the centre and the sub-national units of the state and other organizations within society.

Decentralization is the devolution by central (i.e., national) government of specific functions, with all of the administrative, political and economic attributes that these entail, to local (i.e., municipal) governments which are independent of the central government within a legally delimited geographic and functional domain [23]. De-concentration, delegation, devolution and privatization are the tools of decentralization [24]. Deconcentration is a shift in responsibility from the center to the periphery within an organization [25]. Delegation and devolution reallocates authority in separate government entities or sub-national governments [26]. Privatization is not an independent method of decentralization but implies divestiture while decentralization entails some form of government involvement; however thinly [24].

#### Efficiency

Efficiency is widely used in economics and commonly referred to as the best use of resources in production. Technical efficiency in simpler terms means that material inputs are not wasted. Technical efficiency is considered as ‘the minimum proportion by which a vector of inputs could be reduced while still producing a given output rate’ [27, 28]. For instance, health workers standing idly around, health units waiting for spare parts/instruments/drugs would mean inefficiency. Pure economic efficiency is an unrealistic goal; however, relative efficiency is feasible and measurable [29, 30]. Economic theorists have defined it as the extent to which a given combination of inputs produces as much output as is feasible. There is a general lack of unanimity about how best to measure efficiency. Staff-time utilization is considered a proxy indicator to observe, quantify and analyze efficiency [31]. Therefore, two logical questions emerging from the discussion are: Are some types of facilities more efficient in the production of services than others? Does the efficiency of services improve because of local decision making? Allocative efficiency implies that scarce resources are used in a way that meets the needs of people in an optimal manner. It measures the extent to which an organization is minimizing the cost of producing a desired level of output [32]. In other words, reduction of excess use of inputs would increase technical efficiency and selection of cost-minimizing mix of inputs would lead to allocative efficiency [33]. There is an element of morality inherent in the concept of allocative efficiency. It ensures only that the allocation of resources occur correctly for the wants of the people involved, but not necessarily involve correctness of such decisions - the focus is on consumer satisfaction from available resources. Data Envelopment Analysis (DEA) as a management tool can help the managers to identify sources of cost-inefficiency. Understanding of such concepts and the tools to measure these are critically important from the point of view of local decision makers. Managers at local level and policy makers at strategic level need such ready-to-use information for effective decision making. While assessing the influence of decentralization on performance of health system, funds generation avenues, sectoral allocation of resources, timely availability and utilization of funds need to be investigated.

#### Quality

Quality of services has several meanings, but the two principal dimensions are of access and effectiveness. Quality of health services may be defined as “the degree to which health services for individuals and populations increase the likelihood of desired health outcomes and are consistent with current professional knowledge” [11, 34]. In other words, quality health care should produce desired health outcomes. However, many factors that influence the final outcome of health (e.g., environmental hazards) are beyond the control of individual providers and provider organizations. There are additional notions of equity and safety in it [35]. Quality as a significant dimension of health care may be understood at two different levels: i) as a whole including the resources, the activities, the management and the outcomes of health care - this would imply that quality is the merit or excellence of the system in all its aspects [36]; ii) at a more restricted level it may be considered to be one of the features of the health care resources and activities; for instance, a researcher might just focus to find out if a the health unit complies with the National Accreditation Board of Hospitals (NABH) guidelines? Hence, it may be safe to assume that the outcome of a functional health system ought to depend on the attributes of the resource envelope and activities, while the resource envelope and/or activities need to have an in-built concept of ‘quality’. Yet another aspect of quality of care is the perceived satisfaction of end-users (for instance, patients in hospital settings). We have explained this further in the section of health outcomes, because these are more tangible to the patients than concepts like clinical protocols and non-clinical parameters of health care.

#### Health outcomes

Health outcomes are dependent on the complex interplay of demand- and supply-side factors. However, outcomes may be grouped into four categories: i) h*ealth status:* whether the client is improving, maintaining, or worsening in a hospital or under a community health programme; ii) h*ealth-related knowledge:* what is the level of knowledge and awareness of the client about preventive and curative health; iii) h*ealth-seeking behavior:* the activities that the client initiates for improving and/or maintaining health (e.g., attending a doctor, complying with medication, etc.). Often it is dependent on socio-cultural factors associated with the client; iv) s*atisfaction with care:* the overall experience and degree of satisfaction or dissatisfaction with care received from a health unit (e.g., waiting time, providers’ behavior, etc.). There is a range of dimensions of care that are assessed under existing frameworks, but in recent times outcomes not only reflect the progress and effectiveness of interventions, but also help in monitoring quality improvement [37, 38]. Past studies on performance assessment have measured mainly three types of outcomes: medical outcomes, costs, and client satisfaction. For the last, clients are asked to rate their own health status after receiving care and their satisfaction level with the services delivered [39, 40].

The influence of decentralization and specific outcomes was studied by several scholars. Atkinson and Haran found an association between decentralization and improved performance, but only for 5 of our 22 performance indicators. The study found that good management practices led to decentralized local health systems rather than vice versa. It further concluded that *‘any apparent association between decentralization and performance could be an artefact of the informal management’,* and that *‘the wider political structure strongly influenced the performance of local health systems’* [41].

Faguet examined the influence of decentralization on allocative efficiency in terms of investment patterns and meeting the objective measures of needs across Bolivian municipalities and Spanish provinces and concluded that decentralization had led to a better adjustment between investment patterns and needs [23]. Akin in Uganda analyzed the allocation of funds between public and non-public goods and concluded that the public good activities received lesser resources from regional governments than the central government, implying that social welfare was weakened with decentralization [42]. In another study, Khaleghian used cross-country time series data to assess the effect of decentralization on child immunization and found that decentralized schemes performed better in low-income countries, while the opposite occurred in middle-income countries [43]. Jimenez found similar positive effects of decentralization on health services, analyzing a time series dataset on health spending and child mortality [44]. Another study found fiscal decentralization in the health sector negatively influenced under-five mortality (U5M); on the other hand, fiscal decentralization in provincial revenues improved U5M [45].

Soto et al. analyzed the influence of fiscal decentralization (measured as the locally controlled health expenditure as a proportion of total health expenditure) on health outcomes in Colombia. They concluded Fiscal decentralization decreased infant mortality rates. However, this effect was stronger in non-poor municipalities; however, this *‘depended greatly on the socio-economic conditions of the localities’* [46]. Hotchkiss et al. from Georgia studied the quality and effectiveness of surveillance and public health response in an environment of decentralization. They found improvements in perceived data availability. However, several health system barriers existed that constrained the effectiveness of the intervention in influencing the availability of data, analysis and response [47]. Alejandro and Solé found that road and educational infrastructure investment and capital stocks were sensitive to regional outputs and costs when managed by the local governments [48]. Andrei et al. concluded that in Romania there was no consistently positive effect of decentralization on outputs of public health system [49].

Seitio-Kgokgwe et al. recently evaluated the performance of public hospital system using the World Health Organization Health Systems Performance Assessment Framework (WHO HSPAF). The study concluded that the *‘organizational structure of the Botswana’s public hospital system, authority and decision-making were highly centralized. Overall physical access to health services was high. However, challenges in the distribution of facilities and inpatient beds created inequities and inefficiencies. Capacity of the hospitals to deliver services was limited by inadequate resources’* [50]. Mosquera et al. evaluated the performance of essential dimensions of the primary health centre (PHC) strategy in Colombia, using a rapid assessment tool. They found that the global performance index was rated as good for all interviewees. The weakest dimensions were the family focus and community orientation; the distribution of financial resources; and, accessibility [51].

Wong et al. included inputs, activities, outputs and outcomes in the China Community Health Facilities and Stations (CHS) Logic Model and covered a total of 287 detailed performance indicators to measure performance. The study concluded that a Logic Model framework could be useful in planning, analysis and evaluation of PHC at a system and service level [52]. Topp et al. in Zambian interviewed health workers, patients and directly observed facility operations at PHCs and concluded that the *‘health centre performance was influenced by mechanisms of accountability, which are in turn shaped by dynamic interactions between system hardware and system software’* [53]. Another study by Loveday et al. measured health system performance using a framework of domains comprising key health service components. The authors concluded that the chance of treatment success was greater if decentralized multi-drug resistance (MDR) tuberculosis (TB) services were integrated into existing services [54]. Kalk and Fleischer concluded that *‘Leprosy control in Brazil took advantage of the decentralization process; in Colombia, it came close to a collapse’* [55]. Kolehmainen-Aitken, Riitta-Liisa advocated to define human resource policy, invest in research and identify motivators for retention of health workers [56]. Abimbola et al. studied the influence of decentralization on retention of primary health workers in rural areas in Nigeria and concluded that decentralization delayed the salary payment of health workers, and that the health workers were not keen to work for primary care but opted for secondary tier of care [57].

#### Conceptual approaches

The principal question in the discourse of decentralization in health hinge on whether or not decentralized governance accomplishes stated goals of efficiency, equity and quality of health services [6, 58, 59]. In the absence of conclusive evidences, increasing the depth of ‘decision space’ at local level in financial allocation, organizational design, and human resources deployment may not yield the desired result of drastically improving the performance of peripheral health units. Studies from across the globe have addressed the financial and impact level effects of decentralization mostly through macro analyses, but we came across very limited India-specific empirical studies examining effects of local decision making on health system performance.

Past studies have assessed the extent to which decentralization could serve as a policy instrument for the improvement of a nation’s health system. Mills et al. noted many theoretical benefits of a decentralized health system and concentrated on the gap between the intentions and the reality. They argued that decentralization was never easily implemented and rarely brought immediate gains. They argued, *‘Resistance of civil servants to a change in the power structure, the difficulty of persuading staff and their families to accept peripheral posts, and the risk that greater local authority will mean greater opportunity for patronage and corruption’* as the challenges. Case studies from across the Globe also provide a series of practical and human-related determinants of success of reforms [60].

Tashobya et al. developed a set of attributes for a ‘good’ health system performance assessment (HSPA) framework from and identified key attributes for a HSPA framework. The study advocated for consultative development of a frameworks for health system assessment, as it was found that there existed marked differences between the structure and content of frameworks among countries depending on their per capita income [61]. Savoia et al. developed a conceptual framework to be considered while applying performance measurement science to public health emergency exercises [62].

Kok et al. conducted a systematic review of contextual factors influencing performance of community health workers (CHWs) in low and middle income countries (LMICs). It concluded that research on CHW programs often did not capture the context in which CHW interventions take place, and that future health systems research recognize and address the complexity of contextual influences on programs [63]. Bardhan argued that control rights in governance structures should be placed with local people for better health outcomes [64].

Kogan et al. proposed a three-tiered performance measurement system with national outcome measures (NOMs), national performance measures (NPMs) and evidence-based/informed strategy measures (ESMs) as symbolic of ultimate goals [65]. Anhang et al. examined the association between patient experiences and other measures of health care quality. They concluded that *‘patient experience measures that were collected using psychometrically sound instruments, employing recommended sample sizes and adjustment procedures, and implemented according to standard protocols were appropriate complements for clinical process and outcome measures in public reporting and pay-for-performance’* programs [66]. Tawfik-Shukor et al. undertook an inter-country comparative study of health system performance approaches in The Netherlands with Ontario. The study concluded that both countries differed in their assessment approaches and that *‘several important conceptual and contextual issues must be addressed, before even attempting any comparisons and benchmarking’* [67]. Collins and Green proposed a set of warning questions and issues to be taken into account to ensure that decentralization genuinely facilitates the Primary Health Care orientation of health policy [59].

#### Measuring performance

Measurement functions comprise numerators and denominators; the numerator consists of the population with the characteristic under study, while the denominator contains the target population. Evaluating performance is a significant task [68]. Often the central problem with assessment of performance is lack of reliable data. Studies on performance measurement of decentralization have heavily applied principles of economics and of clinical medicine. For instance, expenditure analysis and per capita spending are used as an indicator of equity [69]. On the other hand, based on clinical medicine parameters, the processes, the immediate results and the outcomes are measured. *Processes:* What activities were carried out to deal with a case (individual) or an episode (community)? *Immediate results:* Was the patient cured? What was the cure rate and mortality rate among patients? *Outcomes:* What changes have taken place in the survival, morbidity, and disability patterns in a given population? This is considered as the final outcome of the health system [70]. But such measurements are generally difficult to carry out and less frequently done.

Tools for measurement of decentralization were studied in different contexts. Veillard J et al. argued that despite the persistence measurement limitations and lack of systematic linkage to decision-making processes, the ministry’s performance management function in Ontario could be strengthened through specific interventions [71]. Gómez in 2003 pointed out that scholars may compare cases based on the horizontal and ex-post vertical political processes of decentralization reform. Second, cases could be compared based on the degree of centre-state policy fluctuation over time. The author encouraged scholars to scale down to the municipal level, comparing cases based on the following variables: historical state-municipal fiscal relations, institutional innovations, and the policy-making process [72]. Balanced scorecard system is advocated for close monitoring of health systems strengthening interventions. In Zambia Mutale et al. found that finance and service delivery domains performed poorly in all study districts. The study found this tool could be valuable in monitoring and evaluation of health systems [73]. Edward et al. used the balanced scorecard (BSC) performance assessments to measure effectiveness, accountability and responsiveness of services in Afghanistan. The study found, among many other key results, the joint interface meeting facilitated transparent dialogue between the community and providers that resulted in creative and participatory problem solving mechanisms [74].

#### Determinants

The determinants of performance are factors related to (i) health workers; (ii) health facilities; (iii) agents of decision making, patients and the community [75]. For instance, in case of health workers, their knowledge and skill-mix, motivation, role clarity and perception of patients’ demands could be critical to determine success of service delivery. Similarly, in health facilities, availability of infrastructure, equipment and supplies, preservation of patients’ rights, accreditation status, and evaluation indicators are important factors. With regard to the ‘agent’ of local decision making (RKS), their role clarity, understanding of local context, knowledge and expertise to plan, prioritize and monitor activities would be of pivotal importance. From the point of view of patients and the community in general, their knowledge about illnesses, health seeking behavior, demand for services, and accessing appropriate care are vital. Measurement of health system performance should include each of these components of multi-dimensional elements that eventually contribute to the overall functioning of health systems.

### Difficulties

#### Challenges in assessment

Assessing the influence of decentralization on health system performance pose four-fold challenges: i) measuring the nature and quantum of local decision making requires mixing a process (nature) with a product (quantity) and the inherent challenges of doing so; ii) suitable blending of macro and micro level analysis is another challenge in measuring efficiency and quality; iii) quality has multi-faceted dimensions and multi-layered elements that act through input-process-output continuum; therefore measurement of quality could envisage reflection of some of its key elements in quantitative terms; and finally, iv) in situations where a reference base-line is not available, which is often the case, the researcher finds it extremely difficult to finalize a suitable evaluation study design. The WHO (2000) has acknowledged the challenges of assessing health systems, assessing outcomes of interest and comparing attainments with what the system should be able to accomplish (performance) [76].

#### Non-feasibility of field trials

If the principal question of investigation is whether or not the performance of the health system improves after introduction of decentralization; and whether there is a cause-effect relationship, one would ideally conduct a randomized controlled trial (RCT). However adopting an experimental study design would not only be costly but also not feasible in most settings. Newer health interventions are constantly designed and implemented by state governments. The damage of depriving a community from such programs would outweigh the benefits of scientific rigor of evidences generated from a classical study design.

#### Difficulty in measuring segmented decentralization

By segmented decentralization we mean decision-making is often mixed among various layers of government. The three basic types (political, fiscal and administrative) of decentralization are often inseparable, and their individual effects on performance couldn’t be evaluated in a segregated manner. For example, decisions related to financial allocation may be centralized, while provision of public services may be decentralized. Taxation and expenditure responsibilities may not be clearly assigned to the central and peripheral governments. The extent to which any particular decision is decentralized may not be clear. Less extensive forms of administrative and fiscal decentralization include deconcentration or mere deployment by the central government of employees to the local level to establish shared governance systems. In all above instances, the researcher invariably comes across methodological challenges in assessing the performance of such systems.

#### Lack of consensus on measurement indicators

Given its various dimensions, measuring decentralization in public health sector in an aggregate manner is a non-linear, complex task. One may apply governance indicators to different layers of government as proxies, but in recent times, more objective indicators describing different aspects of governance have been offered [77–79]. For instance, studies have focused on cross-country indicators as proxies for various aspects of governance, including accountability, political stability, and control of corruption, among others [80]. In principle, each of these aspects can also be applied to decentralized structures, but such governance indicators pose challenges in adjusting for poor coherence and non-comparability of data. Measurement of corruption (based on perceptions), for example, poses difficulties to compare scores between regions. What is considered as an ‘acceptable’ practice by a region might be viewed as a ‘corrupt’ practice in another. Despite these shortcomings, there is fair deal of agreement about the indicator to be used for various research questions. For example, political decentralization may be captured by the tiers of elections; administrative decentralization could be approximated by the degree of sub-division of nation states, and fiscal decentralization could be assessed by the share of sub-national expenditure in total expenditure. However, all these types of proxy indicators have their own deficiencies [81]. A corollary to this is the difficulty in attributing improvement of indicators to a specific health sector reform. Consequently, oftener than not, governments focus on achieving tangible outputs that have strong attributional value than taking a difficult, reformists’ route.

#### Difficulties in measuring quality

Healthcare in general and medical service delivery in particular is dynamic; therefore, quality is ought to be dynamic. Defining quality under such circumstances remains a major challenge. Most definitions rest on two basic concepts: (i) appropriate processes of care; and (ii) patient outcomes or end results of care. The assumption is that when applied properly, the former will maximize the latter [82]. Whereas, other definitions identify the art of care as a function of ‘trust’ between the patient and physician and on humanism [83]. One definition of the “humanistic physician” holds that integrity, respect, and compassion are essential qualities [84, 85]. This underlying assumption that a physician will attempt to do ‘good’ and avoid ‘harm’ is sometimes misplaced. As often is the case in a country like India, where patients are unfamiliar with the larger organizations from which they are seeking care, suspicion and mistrust are frequently reported. There is also rising criticism about the use of clinical outcomes in the evaluation of quality of the care, particularly the mortality rates; it is argued that administrative data do not provide a transparent perspective on quality [86–89]. Finally, the clinical dimensions are themselves expanding, and the definition of quality of health care encompasses the whole of health system, not just the physician alone; health care is increasingly seen as a team effort. Thus, the entire focus on quality of care is getting broadened. This presents a constant challenge as to how we eventually perceive and define overall quality of care.

### Derivatives

#### Demand and supply-side characteristics

**Fig. 2:**
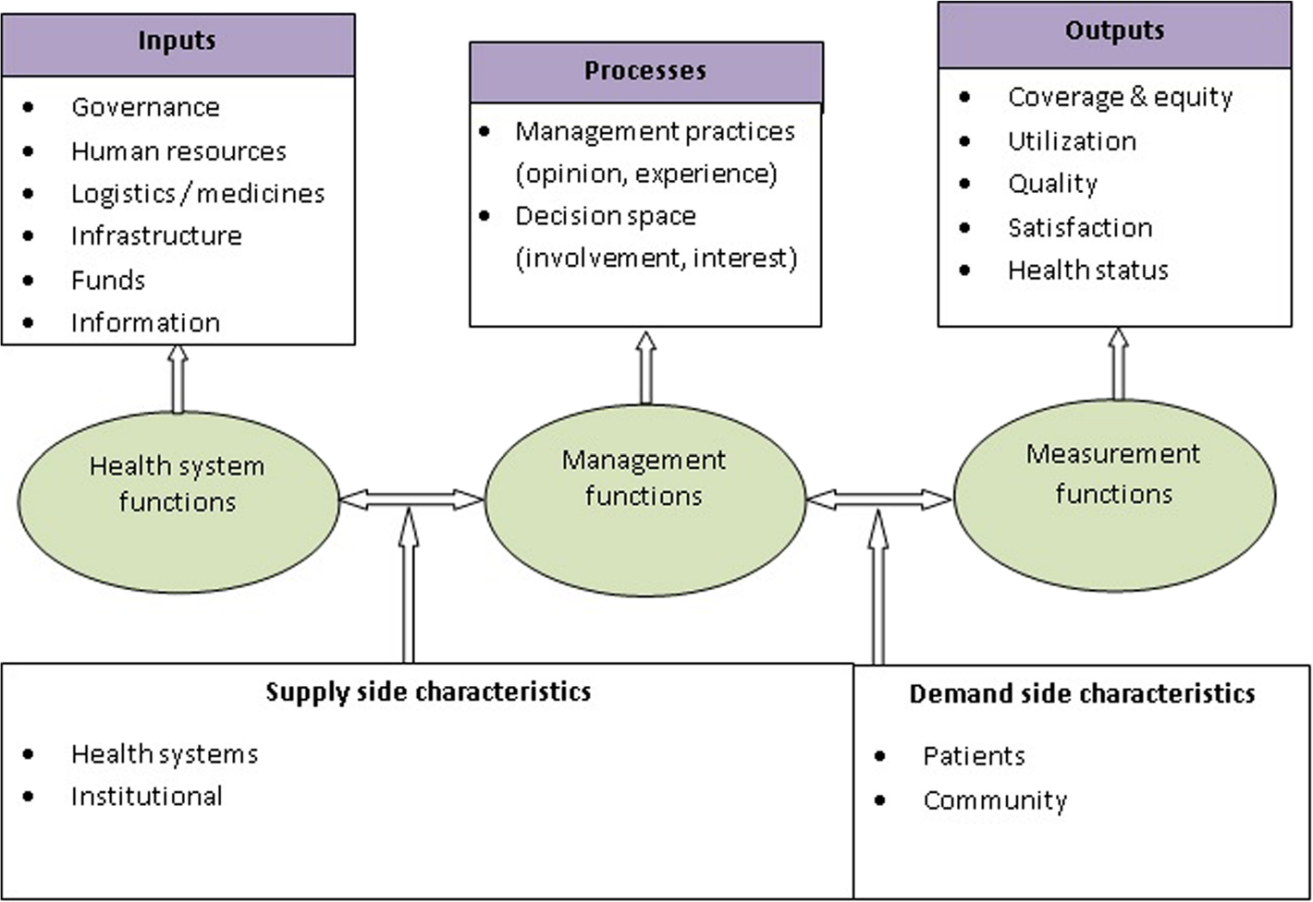
Relationship among inputs, processes and outputs through supply and demand-side factors. Source: Authors’ self-construct, 2015

It is now well accepted that in health sector, there are demand- and supply-side characteristics; both operate through a relatively complex interplay of factors. The locus of control of many such factors is often outside the direct purview of the health department. We have considered the supply side characteristics as i) institutional frameworks and supporting rules and regulations to operationalize institutions; ii) service providers, their knowledge, skills, perceptions and satisfaction; and iii) health system characteristics that are contingent upon the processes being adopted at various levels of the decision making hierarchy. The demand side characteristics on the other hand constitute i) patients who are the principal beneficiaries of the health system; and ii) the community in general (Fig. 2).

#### Input-process-output continuum

An efficient health system is considered as a critical input for success of public health service delivery. Several other ‘inputs’ are necessary to ensure that the health system is not only functional but also responsive to local needs, each of these inputs encompass multiple sub-elements. Compliance of health units with national quality benchmarks, such as, Indian public health standards (IPHS) or international standards organization (ISO) could be construed as symbolic of a well-functioning health system. Hence, study of compliance may be considered as an important question of enquiry in the discourse of decentralization. Institutional processes, such as, management practices (including decision making norms, knowledge, perception and experience of decision makers and service providers) and decision space (including involvement and interest of stakeholders) are critical bridges between the community and the health units as to make the health units responsive. The socio-economic characteristics of patients and the community, their perception, health seeking behavior and satisfaction about the health care services that they receive could serve as critical sub-elements of enquiry at output level. Service utilization is considered as a product of both supply and demand side factors; however, in this paper, we assumed utilization as predominantly a demand side feature.

#### Future enquiry

We propose that assessment of immediate effects of decentralization on health system performance need to dig into the functional areas, elements and sub-elements (Table 1) netted in the discourse. Notwithstanding certain degree of overlapping, the health service delivery system may be categorized at three distinct levels of characteristics - health systems, institutional and community/patients – responsible for three types of functions. Key elements at input level include service providers and the service delivery points. At process level, the elements constitute management practices, while at output level the elements constitute utilization, satisfaction and community participation. The sub-elements (each of which could encompass a number of indicators) against each element may be considered for future scientific enquiry. This framework looks at functions and characteristics through the input-process-output continuum as discussed in the earlier section.

**Table 1:** Levels, functions, elements and sub-elements of enquiry

Level	Characteristics	Functions	Elements	Sub-elements
Input	Health system characteristics	Health system functions	Service providers	• Opinion, experience • Job satisfaction • Authority • Accountability • Competence
			Service delivery point	• Compliance with standards
Process	Institutional characteristics	Management functions	Management environment and practices	• Knowledge, opinion • Policy and guidelines • Role clarity • Competence • Power, accountability
Output	Patient/community characteristics	Measurement functions	Utilization	• Out-door utilization • In-door utilization • Utilization of outreach services
			Satisfaction	• Awareness, perception • Access to services • Satisfaction
			Community participation	• Socio-economic status • Health seeking behavior

Source: Authors’ self-construct, 2016

## Discussion

The building blocks of the health system consist of adequate and ready-to-use infrastructure; availability of a competent and motivated workforce; provision of timely, accurate and reliable information; availability of optimal financial resources; availability of timely and sufficient logistics; and last but not the least, good governance. Allocation and utilization of resources would influence the equity, efficiency and thus quality of services. In Indian context health services are driven predominantly by supply side factors, especially in rural areas. Patients and community members have hardly any voice or note of dissent. Success stories are limited to geographies wherein the local governmental responses are proactive in terms of providing need-based services at the door steps, which are accessible, affordable and acceptable to the general public.

The constitutional provisions envisage a federal structure but unitary spirit, wherein states act as sub-national units, assigned with specified political and fiscal authorities. Further, the constitution gives the central government residual authority and considerable sovereign discretion over the states, implying a relatively centralized federation. The 73^rd^ and 74^th^ Constitutional Amendments of 1992 for the first time realized the importance of devolution of more powers to local self-government institutions - Panchayats for rural areas and Municipalities for urban areas [90]. The impact and experience of such reform has been highly variable, ranging from attempts at *Gram Swaraj* (or village self-rule) in Madhya Pradesh to political recentralization in Karnataka [91]. While sporadic success stories are trickling in, this concept has miles to travel. Interest in health sector reform began in Odisha in the mid-1990s, with introduction of two historic events: (i) the formation of the House Committee of the Orissa Legislature chaired by the Health Minister that dealt with important decisions related to health care, such as user charges, autonomy to major hospitals, and abolition of private practice by government doctors; (ii) the recommendations of the British government’s Department for International Development (DFID) to introduce certain systemic changes in three M’s - maintenance, mobility and medicine [92].

The agenda of local self-governance is taken forward in health sector by formation of functional gaon kalyan samiti (GKS) at village level, rogi kalyan samiti (RKS) at facility level (from PHC onwards to tertiary hospitals) and zilla swasthya samiti (ZSS) at district headquarters level. Stakeholders from diversified departments of the government and elected representatives govern these institutions. As an epitome of shared governance in public health, the RKS undertakes multiple responsibilities and executes a variety of functions aimed at improved accountability and enhanced quality of care, in turn, reduction of mortalities & morbidities through better governance.

Post 2005, major health program investments in India are routed through the national health mission (NHM), in turn, through local self-governing institutions, such as, the (RKS) at all three tiers of service delivery, namely, PHC and community health centre (CHC); district hospital (DH) and medical college hospital (MCH). The ZSS at district (sub regional) level acts as the nodal centre for planning and monitoring of health programs. The RKS is entrusted with the responsibility of planning, monitoring and supervising program implementation of health units, improving quality of services, and ensuring transparency and accountability in decision making. Concepts, such as, efficiency (technical and allocative), determinants of performance, quality of care, health outcomes, and performance measurement are intricately linked with the discourse of decentralization in health.

The key findings from this review indicate that decentralization or local self-governance in public health sector has multiple dimensions in conceptualization, complexities in measurement, and byproducts for consideration. For instance, the capacity of RKS in successful governance of health units would be contingent upon contextual factors, such as, cooperation among members, leadership capabilities, involvement in day-to-day functioning of health units, sense of ownership of the health units, genuine/vested interests of decision makers, and involvement of the community. Weighing different implementation strategies, monitoring and measuring the outputs at facility and community level, and facilitating flow of information to the top would depend upon the willingness on the part of the local self-governing institutions to provide feed-in and receive feedback.

## Conclusion

Evidences from low and middle income countries across the globe indicate that decentralized governance in health across geographic settings could result in a number of possible experiences. Further, concepts of efficiency, accountability, responsiveness, quality of care are difficult to measure in real life settings. There is dearth of studies and evidences from India as to the nature of functioning of RKS as an institution of local self-governance, the perception and experiences of RKS members in governing health units, and the opportunities and barriers to the effective functioning of health units. A central aim of any scientific scrutiny into the effects of decentralization in health should be to examine and assess the role and functions of local decision-making institutions and the results of such decisions at systems, institutions and patient/community level. The vastness of the subject matter of decentralization, diversity of socio-political arrangements, and complex interaction of building blocks of the health system need to be thoroughly acknowledged and properly understood while designing data collection tools for conducting research. Despite the contextual difficulties and methodological challenges, performance measurement at local level from the point of view of decentralization as a health sector reform measure need to examine the health facility-driven processes, community behaviors and providers’ characteristics. Policymakers need to seriously consider the need for investing in capacity building of administrators, local decision makers and service providers as to enable them understand the principles of local self-governance for efficient and effective delivery of healthcare services.

## Study limitations

This review has some limitations. This study included only published literature in PubMed and Google Scholar and excluded literature of other databases. It also excluded grey literature, such as, unpublished reports, media reports, academic theses and conference proceedings. The inclusion of only published literature might have introduced publication bias. It is difficult to establish exclusive objectivity while screening and reviewing articles. To minimize this bias, we used pre-defined inclusion criteria and discussion throughout the review process. Studies which did include India in their analysis could have been missed out.

## References

[1] Anderson G, Hussey PS (2001). Comparing health system performance in OECD countries. Organization for Economic Cooperation and Development. Health Aff (Millwood).

[2] Hafner T, Shiffman J (2013). The emergence of global attention to health systems strengthening. Health Policy Plan.

[3] World Health Organization (2000). The World Health Report 2000 - Health Systems: Improving Performance.

[4] GAVI. GAVI Progress Report 2005. http://www.gavi.org/library/publications/gavi-progress-reports/. Accessed 30 July 2015.

[5] Ooms G, Van Damme W, Baker BK, Zeitz P, Schrecker T (2008). The “diagonal” approach to Global Fund financing: a cure for the broader malaise of health systems?. Global Health.

[6] Heywood P, Choi Y (2010). Health system performance at the district level in Indonesia after decentralization. BMC Int Health Hum Rights.

[7] Haines A, Cassels A (2004). Can the millennium development goals be attained?. BMJ.

[8] Shengelia B, Tandon A, Adams OB, Murray CJL (2005). Access, utilization, quality, and effective coverage: An integrated conceptual framework and measurement strategy. Soc Sci Med.

[9] Sinclair A, Whitford A (2015). Effects of Participation and Collaboration on Perceived Effectiveness of Core Public Health Functions. Am J Public Health.

[10] World Health Organization. Everybody’s business - strengthening health systems to improve health outcomes : WHO’s framework for action. Report. 2007:44. doi: 9789241596077. ISBN 978 92 4 159607 7. http://www.who.int/healthsystems/strategy/everybodys_business.pdf. Accessed 28 July 2015.

[11] Kelley E and JH. “Health Care Quality Indicators Project: Conceptual Framework Paper” OECD Health Working Papers, No. 23, OECD Publishing; 2006. http://www.oecd-ilibrary.org/social-issues-migration-health/health-care-quality-indicators-project_440134737301. Accessed 25 July 2015.

[12] Damanpour FGS (1998). Theories of organizational structure and innovation adoption: the role of environmental change. J Eng Technol Manag.

[13] Pertusa-Ortega EM, Zaragoza-Sáez PC-CE (2010). Can formalization, complexity, and centralization influence knowledge performance?. J Bus Res.

[14] Chegini MG, Yousefi SRS (2013). Competitive Study of Effects about Dimensions of Organizational Structure on Productivity. J Basic Appl Sci Res.

[15] Shadpour K (2006). Health sector reform in Islamic Republic of Iran. Hakim Res J.

[16] Daft RL. Essentials of Organization Theory and Design. South-Western Coll Publ. 1998. ISBN-13: 978-0-324-59889-6 ISBN-10: 0-324-59889-0. http://otgo.tehran.ir/Portals/0/pdf/Organization%20Theory%20and%20Design_1.pdf. Accessed 25 July 2015.

[17] Robbins SP. Organization Theory: Structures, Designs, And Applications, 3/e. Pearson Education India; 1990 Jan.

[18] Irwin A, Valentine N, Brown C (2006). The commission on social determinants of health: Tackling the social roots of health inequities. PLoS Med.

[19] The Free Dictionary by Farlex. Derivative. http://www.thefreedictionary.com/derivative. Accessed 20 July 2015.

[20] Berman PA, Bossert TJ (2000). A Decade of Health Sector Reform in Developing Countries: What Have We Learned?.

[21] Loubiere S, Boyer S, Protopopescu C (2009). Decentralization of HIV care in Cameroon: Increased access to antiretroviral treatment and associated persistent barriers. Health Policy.

[22] Vargas Bustamante A (2010). The tradeoff between centralized and decentralized health services: Evidence from rural areas in Mexico. Soc Sci Med.

[23] Faguet J-P (2004). Does decentralization increase government responsiveness to local needs? Evidence from Bolivia. J Public Econ.

[24] Makara S. Political and Administrative relations in decentralisation. 1998 Decent. Civ. Soc. Uganda. Kampala: Fountain Publishers; 1998.

[25] Rondinelli DA (1983). Implementing decentralization programmes in Asia: a comparative analysis. Public Adm Dev.

[26] Martinez-Vazquez J, McNab RM. Fiscal Decentralization and Economic Growth. SSRN Electron J. 2001. doi:10.2139/ssrn.259281.

[27] Farrell MJ (1957). The Measurement of Productive Efficiency. J R Stat Soc Ser A.

[28] Zieschang KD (1984). An extended farrell technical efficiency measure. J Econ Theory.

[29] Hollingsworth B (2003). Non-Parametric and Parametric Applications Measuring Efficiency in Health Care. Health Care Manag Sci.

[30] Evans DB, Tandon A, Murray CJL, Lauer JA (2001). Comparative efficiency of national health systems: cross national econometric analysis. BMJ.

[31] Perrin B (1998). Effective Use and Misuse of Performance Measurement. Am J Eval.

[32] Rosko MD (1990). Measuring technical efficiency in health care organizations. J Med Syst.

[33] Byrnes P, Valdmanis V (1994). Analyzing Technical and Allocative Efficiency of Hospitals. Data Envelopment Analysis: Theory, Methodology, and Applications.

[34] Lohr KN, Schroeder SA (1990). A strategy for quality assurance in Medicare. N Engl J Med.

[35] Campbell SM, Roland MO, Buetow SA (2000). Defining quality of care. Soc Sci Med.

[36] Donabedian A (2005). Evaluating the Quality of Medical Care. Milbank Q.

[37] Epstein A (1995). Performance reports on quality--prototypes, problems, and prospects. N Engl J Med.

[38] Ben-Zvi S (1989). Quality assurance in transition. Biomed Instrum Technol.

[39] Andrew W (1971). Fisher. Patients’ Evaluation of Outpatient Medical Care. J Med Educ.

[40] Barnett BG (1995). Developing reflection and expertise: can mentors make the difference?. J Educ Adm.

[41] Atkinson S, Haran D (2004). Back to basics: Does decentralization improve health system performance? Evidence from Ceara in north-east Brazil. Bull World Health Organ.

[42] Akin J, Hutchinson P, Strumpf K (2005). Decentralisation and government provision of public goods: The public health sector in Uganda. J Dev Stud.

[43] Khaleghian P (2004). Decentralization and public services: the case of immunization. Soc Sci Med.

[44] Jimenez D, Smith PC (2005). Decentralisation of Health Care and Its Impact on Health Outcomes. Discussion Papers in Economic.

[45] Samadi AH, Keshtkaran A, Kavosi Z, Vahedi S (2013). The Effect of Fiscal Decentralization on Under-five Mortality in Iran: A Panel Data Analysis. Int J Healh Policy Manag.

[46] Soto VE, Farfan MI, Lorant V (2012). Fiscal decentralisation and infant mortality rate: The Colombian case. Soc Sci Med.

[47] Hotchkiss DR, Eisele TP, Djibuti M, Silvestre EA, Rukhadze N (2006). No Title. BMC Public Health.

[48] Esteller A, Solé A (2005). Does Decentralization Improve the Alejandro Esteller and Albert Solé. (2005). Does Decentralization Improve the Efficiency in the Allocation of Public Investment? Evidence from Spain efficiency in the Allocation of Public Investment? Evidence from Spain.

[49] Andrei T, Mitruþ C, Constantin DL, Oancea B (2009). The Impact of Decentralization on Public Health System’ s Results. The Case of Romania. Theor Appl Econ.

[50] Seitio-Kgokgwe O, Gauld RD, Hill PC, Barnett P (2014). Assessing performance of Botswana’s public hospital system: the use of the World Health Organization Health System Performance Assessment Framework. Int J Healh Policy Manag.

[51] Mosquera PA, Hernández J, Vega R, Martínez J, Sebastián M (2013). Performance evaluation of the essential dimensions of the primary health care services in six localities of Bogota–Colombia: a cross-sectional study. BMC Health Serv Res.

[52] Wong ST, Yin D, Bhattacharyya O, Wang B, Liu L, Chen B (2010). Developing a Performance Measurement Framework and Indicators for Community Health Service Facilities in Urban China. BMC Fam Pract.

[53] Topp SM, Chipukuma JM, Hanefeld J (2015). Understanding the dynamic interactions driving Zambian health centre performance: a case-based health systems analysis. Health Policy Plan.

[54] Loveday M, Padayatchi N, Wallengren K (2014). Association between Health Systems Performance and Treatment Outcomes in Patients Co-Infected with MDR-TB and HIV in KwaZulu-Natal, South Africa: Implications for TB Programmes. Caylà JA, ed. PLoS One.

[55] Kalk A, Fleischer K (2004). The decentralization of the health system in Colombia and Brazil and its impact on leprosy control. Lepr Rev.

[56] Kolehmainen-Aitken R-L (2004). Decentralization’s impact on the health workforce: Perspectives of managers, workers and national leaders. Hum Resour Health.

[57] Abimbola S, Olanipekun T, Igbokwe U (2015). How decentralisation influences the retention of primary health care workers in rural Nigeria. Glob Health Action.

[58] Mills A (1994). Decentralization and accountability in the health sector from an international perspective: What are the choices?. Public Adm Dev.

[59] Collins C, Green A (1994). Decentralization and Primary Health Care: Some Negative Implications in Developing Countries. Int J Heal Serv.

[60] Mills A, Vaughan JP, Smith DL, Tabibzadeh I, Organization WH. Health System Decentralization: Concepts, Issues and Country Experience. 1990:146. http://www.who.int/iris/handle/10665/39053. Accessed 25 July 2015.

[61] Tashobya C, da Silveira V, Ssengooba F, Nabyonga-Orem J, Macq J, Criel B (2014). Health systems performance assessment in low-income countries: learning from international experiences. Global Health.

[62] Savoia E, Agboola F, Biddinger P (2014). A Conceptual Framework to Measure Systems’ Performance during Emergency Preparedness Exercises. Int J Environ Res Public Health.

[63] Kok MC, Kane SS, Tulloch O (2015). How does context influence performance of community health workers in low- and middle-income countries? Evidence from the literature. Healh Res Policy Syst.

[64] Bardhan P (2002). Decentralization of Governance and Development. J Econ Perspect.

[65] Kogan MD, Dykton C, Hirai AH (2015). A New Performance Measurement System for Maternal and Child Health in the United States. Matern Child Health J.

[66] Anhang Price R, Elliott MN, Zaslavsky AM (2014). Examining the Role of Patient Experience Surveys in Measuring Health Care Quality. Med Care Res Rev.

[67] Tawfik-Shukor AR, Klazinga NS, Arah OA (2007). Comparing health system performance assessment and management approaches in the Netherlands and Ontario, Canada. BMC Health Serv Res.

[68] Bossert T (1998). Analyzing the decentralization of health systems in developing countries: decision space, innovation and performance. Soc Sci Med.

[69] Parker K, Jacobson A, McGuire M, Zorzi R, Oandasan I (2012). How to build high-quality interprofessional collaboration and education in your hospital: the IP-COMPASS tool. Qual Manag Health Care.

[70] Vuori HV (1982). Quality assurance of health services. Concepts and methodology. Reg Off Eur WHO Copenhagen.

[71] Veillard J, Huynh T, Ardal S (2010). Making health system performance measurement useful to policy makers: aligning strategies, measurement and local health system accountability in ontario. Healthc Policy.

[72] Gómez EJ (2003). Decentralization and municipal governance: Suggested approaches for cross-regional analysis. Stud Comp Int Dev.

[73] Mutale W, Godfrey-Fausset P, Mwanamwenge MT (2013). Measuring Health System Strengthening: Application of the Balanced Scorecard Approach to Rank the Baseline Performance of Three Rural Districts in Zambia. Eisele T, ed. PLoS One.

[74] Edward A, Osei-Bonsu K, Branchini C, Yarghal TS, Arwal SH, Naeem AJ (2015). Enhancing governance and health system accountability for people centered healthcare: an exploratory study of community scorecards in Afghanistan. BMC Health Serv Res.

[75] Rowe AK, De Savigny D, Lanata CF, Victora CG (2005). How can we achieve and maintain high-quality performance of health workers in low-resource settings?. Lancet.

[76] World Health Organization (2000). How Well Do Health Systems Perform? The World Health Report 2000: Health Systems: Improving Performance.

[77] Kaufmann D. Governance Matters VIII Aggregate and Individual Governance Indicators. Policy Res. Work. Pap. [Internet]. 2009;21:1–105. Available from: http://papers.ssrn.com/sol3/papers.cfm?abstract_id=1424591. Accessed 25 July 2015.

[78] Kaufmann D, Kraay A, Mastruzzi M (2011). The Worldwide Governance Indicators: Methodology and Analytical Issues. Hague J Rule Law.

[79] Thomas MA (2010). What Do the Worldwide Governance Indicators Measure?. Eur J Dev Res.

[80] Kaufmann D, Kraay A, Zoido P. Aggregating Governance Indicators. SSRN Electron J. 1999. doi:10.2139/ssrn.188548

[81] Braun JV GU. Does Decentralization Serve the Poor? Cent Dev Res Univ Bonn, Ger IMF-conference Fisc decentralization 20–21 Novemb Washingt DC. 2000. https://www.imf.org/external/pubs/ft/seminar/2000/fiscal/vonbraun.pdf. Accessed 22 July 2015.

[82] Lohr KN, Yordy KD, Thier SO (1988). Current issues in quality of care. Health Aff.

[83] Hall MA, Zheng B, Dugan E (2002). Measuring Patients’ Trust in their Primary Care Providers. Med Care Res Rev.

[84] Linn LS, DiMatteo MR, Cope DW, Robbins A (1987). Measuring Physicians’ Humanistic Attitudes, Values, and Behaviors. Med Care.

[85] Arnold RM (1987). The Humanities, Humanistic Behavior, and the Humane Physician: A Cautionary Note. Ann Intern Med.

[86] Iezzoni LI (1997). The risks of risk adjustment. JAMA.

[87] Best, W., Cowper, R., DianeI C. The Ratio of Observed-to-Expected Mortality as a Quality of Care Indicator in Non-Surgical VA Patients. Med Care. 1994; 32(4). http://journals.lww.com/lww-medicalcare/Abstract/1994/04000/The_Ratio_of_Observed_to_Expected_Mortality_as_a.7.aspx. PubMed PMID: 8139303. Accessed 25 July 2015. 10.1097/00005650-199404000-00007 8139303

[88] Vincent C, Aylin P, Franklin BD (2008). Is health care getting safer?. BMJ.

[89] Iezzoni LI (1997). Assessing Quality Using Administrative Data. Ann Intern Med.

[90] Singh H (1994). Constitutional Base for Panchayati Raj in India: The 73rd Amendment Act. Asian Surv.

[91] Johnson C (2003). Decentralisation in India: Poverty, Politics and Panchayati Raj.

[92] Gupta M (2002). State Health Systems : Orissa. Working Paper No. 89.

